# Grape polyphenols decrease circulating branched chain amino acids in overfed adults

**DOI:** 10.3389/fnut.2022.998044

**Published:** 2022-10-26

**Authors:** Simona Bartova, Francisco Madrid-Gambin, Luis Fernández, Jerome Carayol, Emmanuelle Meugnier, Bérénice Segrestin, Pauline Delage, Nathalie Vionnet, Alexia Boizot, Martine Laville, Hubert Vidal, Santiago Marco, Jörg Hager, Sofia Moco

**Affiliations:** ^1^Nestlé Research, EPFL Innovation Park, Lausanne, Switzerland; ^2^Signal and Information Processing for Sensing Systems, Institute for Bioengineering of Catalonia (IBEC), Barcelona Institute of Science and Technology, Barcelona, Spain; ^3^Department of Electronics and Biomedical Engineering, Universitat de Barcelona, Barcelona, Spain; ^4^University of Lyon, CarMeN Laboratory and Centre de Recherche en Nutrition Humaine Rhône-Alpes (CRNH-RA), INSERM, INRAE, Claude Bernard University Lyon 1, Pierre-Bénite, France; ^5^Service d’Endocrinologie, Diabétologie et Métabolisme, CHU de Lausanne (CHUV), Lausanne, Switzerland

**Keywords:** branched chain amino acids, grape polyphenols, overfeeding, metabolomics, NMR, obesity, human trials, metabolism

## Abstract

**Introduction and aims:**

Dietary polyphenols have long been associated with health benefits, including the prevention of obesity and related chronic diseases. Overfeeding was shown to rapidly induce weight gain and fat mass, associated with mild insulin resistance in humans, and thus represents a suitable model of the metabolic complications resulting from obesity. We studied the effects of a polyphenol-rich grape extract supplementation on the plasma metabolome during an overfeeding intervention in adults, in two randomized parallel controlled clinical trials.

**Methods:**

Blood plasma samples from 40 normal weight to overweight male adults, submitted to a 31-day overfeeding (additional 50% of energy requirement by a high calorie-high fructose diet), given either 2 g/day grape polyphenol extract or a placebo at 0, 15, 21, and 31 days were analyzed (Lyon study). Samples from a similarly designed trial on females (20 subjects) were collected in parallel (Lausanne study). Nuclear magnetic resonance (NMR)-based metabolomics was conducted to characterize metabolome changes induced by overfeeding and associated effects from polyphenol supplementation. The clinical trials are registered under the numbers NCT02145780 and NCT02225457 at ClinicalTrials.gov.

**Results:**

Changes in plasma levels of many metabolic markers, including branched chain amino acids (BCAA), ketone bodies and glucose in both placebo as well as upon polyphenol intervention were identified in the Lyon study. Polyphenol supplementation counterbalanced levels of BCAA found to be induced by overfeeding. These results were further corroborated in the Lausanne female study.

**Conclusion:**

Administration of grape polyphenol-rich extract over 1 month period was associated with a protective metabolic effect against overfeeding in adults.

## Introduction

Obesity is a rising medical condition with >650 million adults worldwide considered obese (BMI ≥ 30) by the World Health Organization (1). Obesity is a major risk factor in the development of various metabolic conditions, such as cardiovascular disease, type II diabetes, dyslipidemias, non-alcoholic fatty liver disease, Alzheimer’s disease and certain cancers (2). When energy intake exceeds energy expenditure over prolonged periods, then an obesity phenotype can develop. The imposition of a short-term positive energy balance can be achieved by an overfeeding intervention. Overfeeding was shown to induce in a few days a significant accumulation of fat mass, both subcutaneously and viscerally, and a transient deterioration of insulin sensitivity markers in humans, and thus represents a suitable model of the metabolic complications resulting from weight gain (3–5).

Current treatments for obesity include reduction of energy intake, increase in energy expenditure or both. There are still limited options in terms of drug therapies, with only a handful of FDA-approved drugs specifically targeting weight loss (6, 7). In cases of extreme obesity, bariatric surgery may be recommended (2). However, in many cases diet and exercise will be effective in overweight individuals, as ways of reducing energy intake and increasing energy expenditure, respectively. Wholesome diets, rich in fruit and vegetables are considered healthy and contain high amounts of polyphenols (8). In general, dietary polyphenols have long been associated with health benefits, including reduction of incidence of cardiovascular events (9), improvement of postprandial glucose (10), and prevention of obesity (11–13).

A non-invasive way to monitor disease- or diet-induced metabolic alterations is to perform metabolomics analyses on human biofluids, such as plasma, within clinical studies (14). Nuclear magnetic resonance (NMR) is particularly useful in studying metabolic effects through metabolomics analyses (15). Thus, altered metabolomes have been reported in metabolic conditions such as obesity (16), as well as in polyphenol-rich diets, like the Mediterranean diet (17).

In this study, we aimed to monitor the metabolic effects induced by polyphenol supplementation on an overfeeding intervention in adults for 31 days. Plasma samples from 2 randomized parallel controlled clinical trials conducted either on males (Lyon study) or on females (Lausanne study) were collected for metabolomics analyses. The clinical findings of these 2 studies have been previously reported in which metabolic alterations were identified both due to overfeeding, as well as due to polyphenol intake (18).

## Materials and methods

### Study design

**FIGURE 1:**
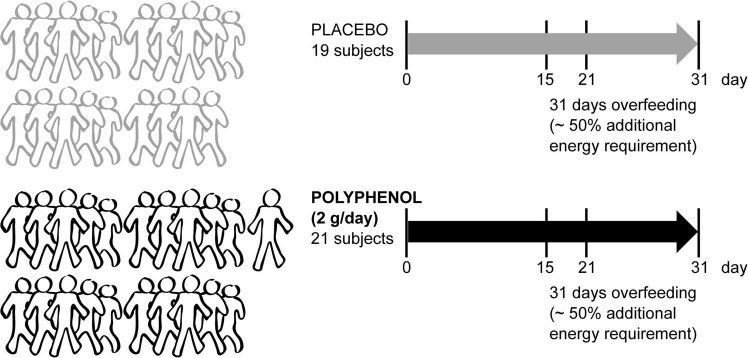
Metabolomics blood sample analysis of the male “Lyon” human clinical intervention. Forty-two sedentary male adult subjects were recruited to a randomized parallel-controlled trial where all subjects were submitted to an overfeeding intervention for 31 days with ∼50% additional energy requirement. Half of the subjects took a placebo, while the rest were given 2 g/day of polyphenol-rich grape extract. The fasting blood collection days are indicated (0, 15, 21, and 31 days). Plasma metabolomics was performed in samples from 40 subjects (19 placebo and 21 polyphenol).

A total of 42 male volunteers were recruited for the “Lyon trial,” registered at ClinicalTrials.gov under NCT02145780 “Polyphenols and Overfeeding (Poly-Nut)” in Lyon, France. Written informed consent was obtained from all participants and approved in Lyon by the ethics committee of Lyon Sud-Est. In short, all adult individuals (males) were submitted to a 31 day-overfeeding intervention (high calorie-high fructose overfeeding protocol, with a daily energy excess of +50% of the total energy expenditure, provided by ultra-processed food such as soda, chocolate breads, chips and chocolate bars to mimic the western diet), including 4 visits in which fasting blood was collected (0, 15, 21, and 31 days) (Figure 1). The intervention consisted of 2 arms (21 subjects in the polyphenol group and 21 subjects in the placebo group), in addition to the overfeeding. Polyphenol supplementation consisted of 2 g/day of total polyphenols [polyphenol-rich grape extract with 20% (m/m) of (pro)anthocyanidins (18)]. Blood plasma was collected and taken for metabolomics analysis from 40 subjects (19 subjects in placebo and 21 in polyphenol groups) attending 4 visits.

A parallel study with an analogous design was conducted in Lausanne, Switzerland, the “Lausanne study,” registered at ClinicalTrials.gov under NCT02225457 “Study of the Mechanisms of Metabolic Adaptations to Overfeeding.” From the “Lausanne study,” 2 arms (women under placebo or polyphenol) were analyzed in this study (Supplementary Figure 1). Blood plasma was collected and taken for metabolomics analysis from 20 female subjects: 9 subjects under placebo and 11 subjects under polyphenol administration (except for T0, where only 8 and 10 samples, respectively, were available). The protocol for this trial was approved in Lausanne by the regional committee for human experimentation (Vaud, Switzerland). Basic baseline clinical parameters of the two studies were included in Supplementary Table 1. Further details, including clinical chemistry, diet details and other parameters were described previously (18).

### Plasma nuclear magnetic resonance-based metabolomics

Blood plasma heparin samples were shipped in dry ice from the trial sites in Lyon and Lausanne to Nestle Research and stored at −80^°^C prior to analysis. Plasma samples were randomized, extracted and analyzed in a 600 MHz Bruker Avance III NMR spectrometer equipped with a 5 mm TCI cryogenic probe. The ^1^H NMR experiment used for acquiring metabolomics data was 1H NOESY-1D (noesygppr1d; 90° pulse is applied with a relaxation delay of 4 s and acquisition time of 2.73 s; the 90° pulse and the pre-saturation power is adjusted automatically for each sample; receiver gain 32; spectral width 30 ppm; 98 k time domain). The number of scans was optimized to 128, with 4 initial dummy scans, leading to a total of 14 min 53 s of acquisition time per sample. More details are described in Supplementary Methods.

### Statistical analyses

For exploratory purposes, the area under the curve (AUC) of the receiver operating characteristic (ROC) curve was evaluated using a double cross-validation technique (dCV) (19) on the preprocessed full NMR spectra at the different study timepoints to explore prediction capability of the intervention groups. Subsequently, NMR spectra were processed using the AlpsNMR R package (20) for automated signal detection and integration. After obtaining the peak table, selected metabolites were integrated for targeted analysis (Supplementary Table 2), and a wider metabolite selection was utilized for correlation analyses. Univariate (paired) *t*-test analyses and visualization on this subset were performed using GraphPad Prism 7.02. Metabolite correlation matrices were obtained using the R package *corrplot*. For untargeted machine learning studies, data were normalized using probabilistic quotient normalization (PQN) (21) and Pareto scaling. To evaluate the differences between the polyphenol and placebo intervention groups predictive multivariate models based on Partial Least Squares Discriminant Analysis (PLS-DA) with feature selection based on recursive ranking based on Variable Importance in Projection were built (22). A multilevel modality of PLS-DA was utilized to assess differences between timepoints of each intervention group. Finally, the statistical significance of the final performance was assessed with a permutation test. More details are found in Supplementary methods.

## Results

**FIGURE 2:**
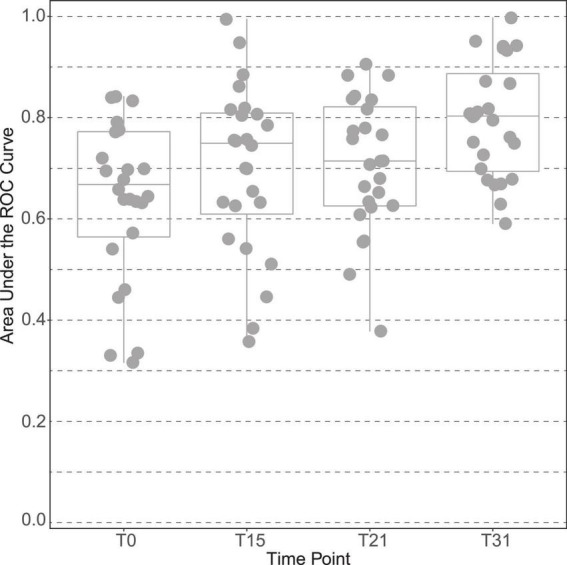
Discrimination capacity of intervention groups across time points of the male “Lyon” study. The plot is based on the area under the curve of the receiver operating characteristic (ROC) curves between placebo and polyphenol groups at all-time points of the intervention: time point 0 (T0), at 15 days (T15), at 21 days (T21) and at the end, time point 31 days (T31).

The plasma of male subjects collected at 4 time points along the interventions (baseline, T0; day 15, T15; day 21, T21, and end of trial at day 31, T31) were analyzed by ^1^H NMR metabolomics. Spectral exploratory analysis showed discrimination capability between treatments that increased with time of exposure (Figure 2). Among all visits, the highest classification performance was observed after 1 month of study (T31), in which participants accumulated the largest exposure to the intervention.

### Overfeeding increased circulating branched chain amino acids in male subjects

**FIGURE 3:**
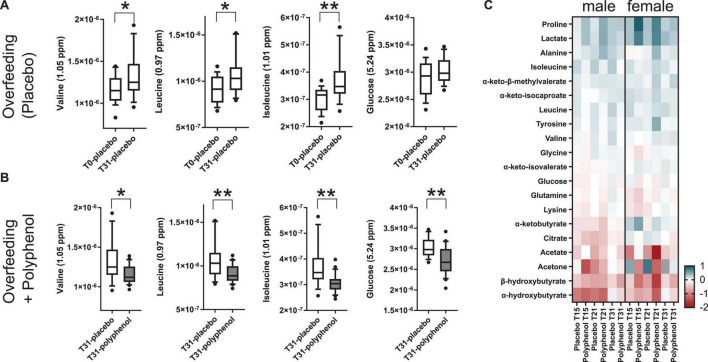
Relative plasma metabolite concentrations of highlighted metabolites during an overfeeding and polyphenol clinical interventions for the male “Lyon” study **(A–C)** and the female “Lausanne” study **(C)**. **(A)** Overfeeding (placebo arm) in males (Lyon study) leads to increased plasma concentrations of the BCAA valine, leucine and isoleucine and glucose. **(B)** A 31-day polyphenol administration in an overfeeding intervention (polyphenol arm) in males (Lyon study) leads to decreased plasma concentrations of the BCAA valine, leucine and isoleucine and glucose compared to the overfeeding arm. **(C)** Heatmap of log2 fold changes of plasma metabolite relative concentrations, adjusted per each individual’s baseline (T0), in the male (Lyon) and female (Lausanne) studies. T0, T15, T21, and T31 correspond to days 0, 15, 21, and 31 of the intervention; the NMR intensities relate to metabolite concentrations (arbitrary units); *p*-values obtained by *t*-test (*p-value ≤ 0.05, and **p-value ≤ 0.01).

**FIGURE 4:**
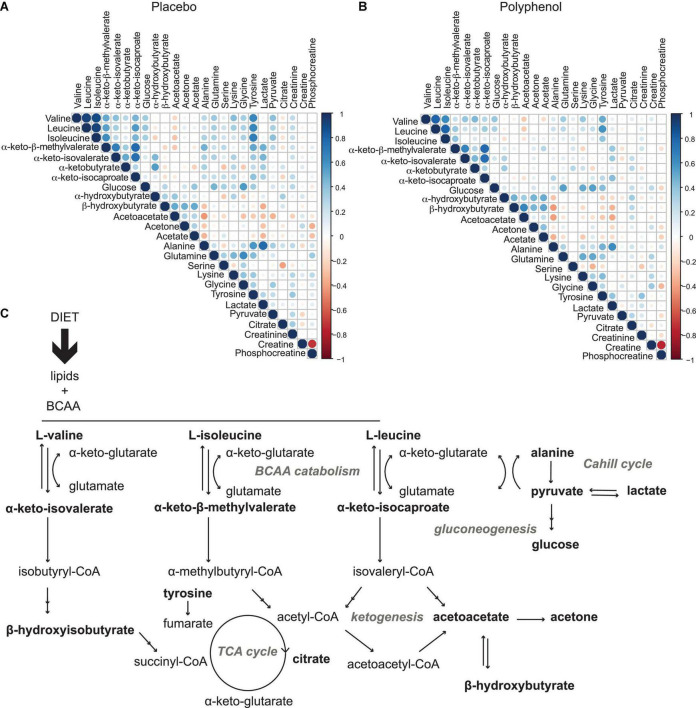
Metabolic effects induced by overfeeding and polyphenol administration. **(A)** Metabolite correlation matrix obtained from the plasma metabolome analysis of the male “Lyon” study across all time points for all subjects for the placebo arm (overfeeding), and **(B)**, for the polyphenol arm (overfeeding and polyphenol administration). Non-significant correlations (*p*-value > 0.05) were excluded. **(C)** metabolic pathways involved in diet-induced obesity, as observed in this study by plasma metabolomics: a profile with elevated circulating branched chain amino acids, BCAA (valine, isoleucine and leucine) and branched chain keto acids, BCKA (α-keto-isovalerate, α-keto-β-methylvalerate, and α-keto-isocaproate) highlighting increased BCAA catabolism, leading to decreased TCA cycle activity (lower levels of citrate) and ketogenesis (lower levels of ketone bodies acetone, acetoacetate and β-hydroxybuturate), with an increased gluconeogenesis (higher levels of glucose) and lactate production through the Cahill cycle. Monitored metabolites are in bold. Pathway names are in gray.

To explore the driving metabolic features behind the overfeeding arm of the intervention (placebo), both multilevel PLS-DA was performed on the full dataset, as well as univariate statistics on integrated metabolites, comparing the last time point of the intervention (T31) to baseline (T0). Levels of circulating BCAA valine (*p*-value 1.8 × 10^–2^), leucine (*p*-value 2.6 × 10^–3^), isoleucine (*p*-value 2.3 × 10^–5^) and the branched chain keto acid (BCKA) α-keto-β-methylvalerate (keto-isoleucine) increased with overfeeding (Supplementary Tables 3, 4 and Figure 3A). Levels of glucose showed a non-significant tendency to increase at the end of the intervention (Figure 3A). Upon overfeeding, most metabolites (amino acids and keto-acids) increased their circulating concentration, while certain ketone bodies decreased, such as β-hydroxybutyrate (Figure 3C). There was a pronounced decrease during the first two timepoints on the overfeeding regime, T15 and T21, that was not significant at T31. The metabolite correlation matrix (Figure 4A) showed positive correlations between BCAA, keto acids and tyrosine. Lactate and alanine, two metabolites that increased along the intervention, seemed also to positively correlate to each other. These two metabolites negatively correlate with several ketone bodies, as β-hydroxybutyrate, acetoacetate and acetone. Acetoacetate also had a negative correlation with BCAA.

### Polyphenol consumption compensated markers of overfeeding in male subjects

Polyphenol consumption under an overfeeding regime led to few differential metabolites, such as alanine and β-hydroxybutyrate at the end of the intervention (Supplementary Tables 5, 6 and Figure 3C). In general, the effect of polyphenol consumption while on an overfeeding regime modulated the metabolome in a similar way to the overfeeding arm. However, at day 31, the polyphenol treated group showed significantly reduced circulating levels of all BCAA compared to placebo (Supplementary Table 6 and Figure 3B). Whereas plasma glucose levels didn’t significantly change at the end of intervention compared to its baseline (T0/T31) for placebo as well as polyphenol arm (Supplementary Table 4), the glucose levels were significantly decreased in the polyphenol group at days 15, 21, and 31 compared to placebo (Supplementary Table 6 and Figure 3B). The amino acids alanine and proline were significantly increased in the polyphenol arm (Figure 3C and Supplementary Table 4). Milder but analogous correlations between metabolites were found upon polyphenol intervention compared to those in the placebo group (Figure 4).

### Polyphenol consumption remodels the plasma metabolome of female subjects

The results of the male study were further confirmed in an analogous human trial conducted in female subjects, the “Lausanne study.” The plasma of female subjects collected at 4 time points along the placebo and polyphenol interventions (baseline, T0; day 15, T15; day 21, T21, and end of trial at day 31, T31) were analyzed by ^1^H NMR metabolomics. The same panel of metabolites used in the Lyon study (Supplementary Table 2), was integrated in this dataset. Mostly non-significant changes were observed along the course of the intervention with a few exceptions (Supplementary Table 7). The overall tendency in plasma metabolite fold changes (Figure 3C) were comparable to the ones obtained in the male trial. The levels of BCAA were increased by overfeeding in both trials. However, in the male study, the levels of BCAA were counterbalanced by polyphenol administration, leading to decreased circulating values (Figures 3A–C); while in the female study, only isoleucine was found to decrease by polyphenol intervention (Figure 3C). In both studies, there was a significant decrease of the ketone body β-hydroxybutyrate by overfeeding at T21 (females *p*-value 2.5 × 10^–2^ and males *p*-value 4.3 × 10^–3^) and an increase in lactate in the polyphenol groups, both at T15 and T21 (males: *p*-value 7.3 × 10^–3^ at T15, 1.6 × 10^–2^ at T21, females: 2.3 × 10^–2^ at T15, 2.5 × 10^–3^ at T21 compared to T0 (Supplementary Tables 4, 7 and Figure 3C). A decrease of plasma glucose levels was observed in both studies upon polyphenol intervention (Figure 3C).

## Discussion

In overfeeding, a surplus of sugar and fat were supplemented in the male and female trials, leading to the subjects’ weight and fat mass gain at the end of the intervention in both placebo and polyphenol groups, as previously reported (18). Furthermore, overfeeding didn’t induce changes in fasting glycemia, but it induced an increase in fasting insulinemia. Total cholesterol and HDL-cholesterol increased in both males and females at the end of the nutritional intervention. While polyphenol supplementation by 2 g/day of grape extract led to increased levels in several polyphenol metabolites at day 28 in the polyphenol group only, it did not modify the clinical parameters measured or counteracted the changes during overfeeding, neither in men nor in women (18).

In this study, however, NMR-based metabolomics allowed obtaining a more detailed picture at the biochemical level, of the nutritional effects of overfeeding in males and females. Pronounced metabolic changes along a month-long overfeeding and polyphenol intervention were observed in the subjects’ plasma. Using the strategy described here, a metabolic profile composed by various metabolites, as organic acids, amino acids, and other small molecules was obtained, reflective of altered metabolism.

Given the excessive intake of nutrients, levels of many metabolites increased in circulation (Figure 3C). Lactate and pyruvate, both products of glycolysis, increased probably due to an insufficient capacity of the mitochondria in tissues to take in pyruvate to utilize in the TCA cycle (Figures 3C, 4A,C). The elevated levels of lactate, pyruvate and alanine, suggest interplay between liver and skeletal muscle through the blood, known as the Cahill cycle (23). To note is that, in accordance to previous findings (18), circulating glucose was also not significantly affected by overfeeding (Figure 3). As the diet was rich in lipids, there is an excess of lipids to be catabolized in the mitochondria, contributing to an assumed mitochondrial dysfunction. As mitochondria become impaired, other processes such as ketogenesis were repressed, leading to decreased levels of ketone bodies (acetone, acetoacetate and β-hydroxybutyrate) in circulation (Figures 3C, 4A). A pronounced BCAA and BCKA profile was found after a month of overfeeding (Figure 3A). This finding is in accordance with previous reports, where elevated BCAA associate with metabolic deregulated status and conditions, including obesity (16, 24). The catabolism of BCAA is distributed in different organs throughout the body, rather than being confined in the liver (25). BCKA, obtained by transamination of BCAA, are normally low in tissues, assumedly due to high concentration of glutamate favoring the re-amination of BCKA to BCAA. In organs and tissues throughout the body, nitrogen from BCAA is incorporated into alanine and glutamine, released in the blood. As a result, metabolism of carbon and nitrogen backbones of BCAA involves inter-organ relationship. High BCAA in circulation, especially leucine, can influence insulin release, it can influence tissue protein synthesis and catabolism, as well as the catabolism of other BCAA. Thus, both BCAA and BCKA exert regulatory effects (26). In addition to BCAA, the amino acids glutamine and tyrosine have also been reported as biomarkers of obesity (27), which seem to also increase in the Lyon study along the overfeeding intervention (Figures 3C, 4A).

Polyphenols supplementation did not significantly counterbalance any clinical parameters induced by overfeeding (18). Interestingly, our metabolomics studies suggest that polyphenol administration might however have a protective effect against overfeeding in men (Lyon study). This is observed by decreased levels of BCAA and glucose, compared to control (overfeeding) in the end of intervention (Figures 3B,C, 4B,C). A similar phenotype was observed in women in the Lausanne study. While this female study contained fewer subjects, it highlighted similar metabolic trends, suggesting a common metabolic adaptation in counterbalancing an obesity phenotype by polyphenol intake. Polyphenols have been extensively studied for their content in foods, bioavailability and metabolic fate, mechanism-of-action and metabolic effects (8, 28–30). In human interventions, polyphenol intake was inversely correlated with BMI in European adolescents in the HELENA study (31). An improved lipoprotein and lipid profiles in overweight women was obtained upon polyphenol consumption (32). In particular, grape polyphenols induced lower plasma levels of LDL-C and ox-LDL-C (33). A Mediterranean diet rich in extra-virgin olive oil significantly reduced levels of BCAA and attenuated the positive association between plasma BCAA levels and type 2 diabetes incidence in the PREDIMED trial (17). In the TOSCA.IT study, polyphenol intake was associated with a better cardiovascular risk factor profile (34). However, polyphenol intake does not always associate to improved metabolic markers. In fact, the extensive metabolism of polyphenols, including by the gut microbiota, induces a large inter-individual variability (35).

Whereas classical clinical parameters didn’t reveal any striking effect of polyphenol intervention during overfeeding in the present trials (18), the metabolomics approach showed several mild changes that are potentially early markers of a metabolic change, possibly not yet detectable by classical approaches after 1 month-long treatment. In sum, using plasma metabolomics, inter-organ metabolic changes could be observed reflective of an obesity-like phenotype, induced by overfeeding. In males and females, an improved metabolic profile was observed upon polyphenol administration, suggesting potential health benefits of a polyphenol-rich diet or supplementation in an overfeeding regime.

### Strengths and limitations

The main strength of this study is the identification of metabolic markers of overfeeding and polyphenol consumption, using a metabolomics approach. These changes were not revealed by classical clinical measurements (18). Therefore, we see potential in such approach in the early detection of nutritional metabolic markers. In terms of limitations, this study could have profited from the integration of additional datasets such as: polyphenol metabolites in circulation, and/or microbiota composition. Whilst interesting findings were here reported, a more mechanistic insight could only be achieved with complementary testing.

## Data availability statement

The original contributions presented in this study are included in the article/Supplementary material, further inquiries can be directed to the corresponding author/s.

## Ethics statement

The protocol was approved in Lyon by the Ethics Committee of Lyon Sud-Est according to the French “Huriet-Serusclat” law, and in Lausanne by the Regional Committee for human experimentation (Vaud, Switzerland). All participants were given oral and written information before the protocol, and a written informed consent was obtained from all of them. The patients/participants provided their written informed consent to participate in this study.

## Author contributions

SB and SoM conducted the metabolomics analysis, its interpretation, and wrote this manuscript. FM-G, LF, JC, and SaM conducted statistical analyses. EM, BS, PD, NV, AB, ML, HV, and JH conducted the clinical trials. All authors contributed to the article and approved the submitted version.
