# Supplementary material for: Grape polyphenols decrease circulating branched chain amino acids in overfed adults
Source: Front Nutr. 2022 Oct 26;9:998044. doi: 10.3389/fnut.2022.998044 (PMC9643885; doi:10.3389/fnut.2022.998044)
Supplement: Supplementary file 1 [file Data_Sheet_1.pdf]

Grape polyphenols decrease circulating branched chain amino acids in overfed adults

Bartova et al

SUPPLEMENTARY MATERIALS

Supplementary Methods

Supplementary NMR-based metabolomics

A blood plasma heparin samples were shipped in dry ice from the trial sites in Lyon and Lausanne to Nestle Research and stored -80 ^{o}C prior to analysis. Before analysis, all samples were randomized before proceeding with sample preparation. During sample preparation, the samples were kept in ice to prevent degradation. To an extraction mixture of 1200 µL of cooled methanol, 2.1 µL of 500 mM maleic acid (Sigma-Aldrich TraceCERT®) and 400 µL of plasma were added. Maleic acid served as an internal standard to control the extraction quality. The extracts were placed at -80 °C for 30 min and centrifuged at 4 °C, 3220 rcf for 15 min. Supernatants were dried overnight in a centrifugal freeze-dryer. The dried pellets were dissolved in 600 µL of 0.2 M PBS buffer (phosphate buffer saline pH 7.40 with 0.07 g/L TSP (3-(trimethylsilyl)-2,2',3,3'-tetradeuteropropionic acid sodium salt) and 0.2 g/L sodium azide in D2O), and 550 µL of this solution was transferred into 5 mm NMR tubes.
NMR spectral acquisition was performed on a 600 MHz Bruker Avance III NMR spectrometer equipped with a 5 mm TCI cryogenic probe. The temperature was fixed to 300 K for all NMR measurements. The ^{1}H NMR experiment used for acquiring metabolomics data was 1H NOESY-1D (noesygppr1d; 90° pulse is applied with a relaxation delay of 4 s and acquisition time of 2.73 s; the 90° pulse and the pre-saturation power is adjusted automatically for each sample using the pulsecal algorithm; receiver gain 32; spectral width 30 ppm; 98k time domain). The number of scans was optimized to 128, with 4 initial dummy scans, leading to a total of 14 min 53 s of acquisition time per sample. Spectra were processed using the automated program apk0.noe, which includes Fourier transformation with exponential multiplication, zero order phase correction, baseline correction, and calibration of the chemical shift axis to TSP = 0 ppm. All NMR spectral acquisition and pre-processing were done under the control of TopSpin 3.5 pl6 (Bruker BioSpin), and the automated submission of a sequence of samples was performed using ICON-NMR 5 (Bruker BioSpin). For a few samples, 2D NMR spectra were recorded, namely [^{1}H,^{1}H]-COSY (Homonuclear Correlation Spectroscopy, cosygpprqf), [^{1}H,^{1}H]-TOCSY (Total Correlated Spectroscopy, mlevphpr.2) and [^{1}H,^{13}C]-HSQC (Heteronuclear Single-Quantum Correlation, hsqcedetgpsp.3). The 2D NMR experiments together with the BBIOREFFCODE database (Bruker BioSpin), the spectral database of small molecules and metabolites, the Human Metabolome Database (HMDB)(1) and literature information allowed for NMR signal assignment into metabolite names.

Supplementary Statistical Analyses

NMR spectra were modelled using our in-house implemented NMR pipeline, based on the AlpsNMR R package (2) for automated signal detection and integration. AlpsNMR includes spectra loading, metadata handling, automated outlier detection, spectra alignment and

peak-picking, integration, and normalization. The following parameters were used: interpolation min 0.2, max 10, by 0.00023; signal-to-noise ratio threshold 0.3; maximal shift 0.0015 ppm; normalization PQN (probabilistic quotient normalization)(3). To evaluate the performance of the predictive models at the different timepoints a double cross-validation technique (dCV) was used (4). The area under the curve (AUC) of the receiver operating characteristic (ROC) curve on external validation was used as a figure of merit. After performing the differences between Pareto-scaled data from baseline and day-31 of each volunteer, multivariate predictive models including Partial Least Square Discriminant Analysis (PLS-DA) and multilevel PLS-DA were applied on differential data (5). First, PLS-DA was used to assess differences between polyphenol and placebo groups on day-31 baseline-subtracted samples. Then, multilevel PLS-DA for paired comparisons was used to separate within-subject variation from the inter-subject variation. This approach was applied on paired samples from day-31 and baseline for each group (placebo and polyphenol) separately. The modelling was performed in a repeated double cross-validation (rdCV) framework optimized for unbiased variable selection using the "MUVR" R-package version 0.971 (6).

Supplementary Figures

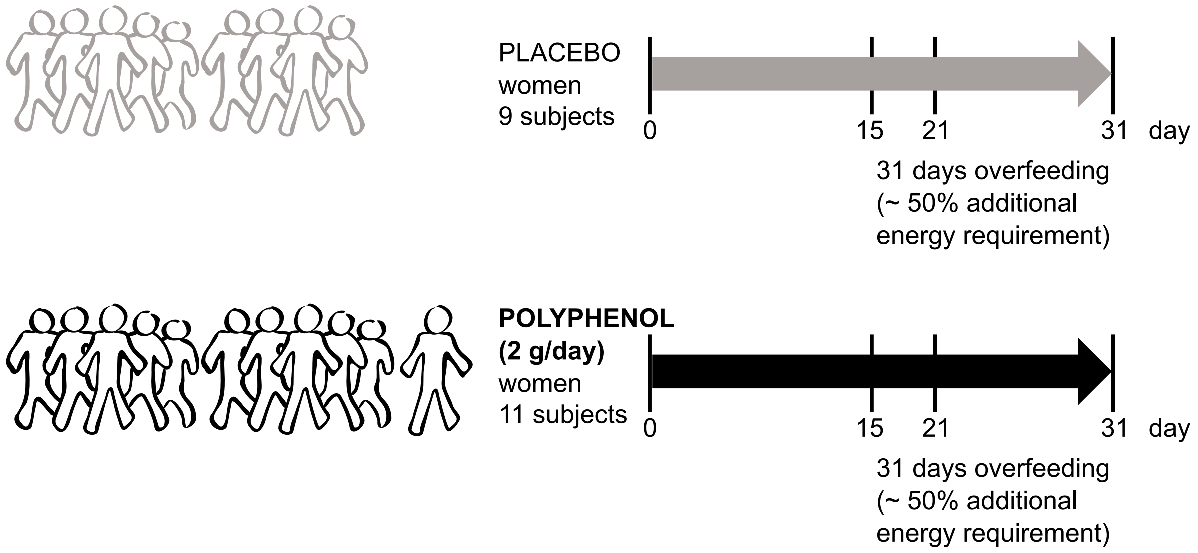

Figure 1S. Metabolomics blood sample analysis of the male 'Lausanne' human clinical intervention.
Twenty sedentary female adult subjects were recruited to a randomized parallel-controlled trial where all subjects were submitted to an overfeeding intervention for 31 days with ~ 50% additional energy requirement. Half of the subjects took a placebo, while the rest were given 2 g/day of polyphenol-rich grape seed extract. The fasting blood collection days are indicated (0, 15, 21 and 31 days). Plasma metabolomics analysis was performed in samples from 20 female subjects (9 placebo and 11 polyphenol, except for T0, where only 8 and 10 samples, respectively, were available).

Supplementary Tables

Table 1S. Baseline clinical parameters of the enrolled subjects in the Lyon and Lausanne studies taken from (7). Data are expressed as mean ± SEM.

	Placebo			Polyphenol		
Lyon study						
Men (n=)	21			21		
Age (y)	33	±	2	29	±	2
Body Mass index (kg/m^2)	25.1	±	0.3	24.8	±	0.3
Body weight(kg)	79.8	±	1.6	80.1	±	1.6
Fat mass(kg)	19.6	±	1.2	21.2	±	1.2
Lausanne study						
Women (n=)	9			11		
Age	24	±	1	27	±	1
Body Mass index (kg/m^2)	20.9	±	0.5	21.8	±	0.6
Body weight(kg)	59.6	±	2.2	60.3	±	3.0
Fat mass(kg)	16.5	±	1.1	19.4	±	2.0

Table 2S. List of metabolites corresponding to indicated spectral regions in ^{1}H NMR spectra.

Metabolite	δ_H (ppm)
glucose	5.24
lactate	4.12
citrate	2.67
acetate	1.92
acetone	2.24
β-hydroxybutyrate	2.39
α-hydroxybutyrate	0.90
acetoacetate	2.28
alanine	1.49
proline	4.14
glycine	3.57
lysine	3.02
glutamine	2.46
serine	3.98
pyruvate	2.37
creatinine	4.06
creatine	3.04
Phosphocreatine	3.05
tyrosine	7.20
valine	1.00
leucine	0.97
isoleucine	1.01
α-keto-isocaproate	2.62
α-keto-β-methylvalerate	1.10
α-keto-isovalerate	1.13

α-ketobutyrate	1.07

Table 3S. Discriminant plasma metabolites after the exposure to 31 days of overfeeding (placebo, T31 versus T0) obtained by multi-level PLS-DA (model with a classification rate of 0.894 (95% CI of 0.752 and 0.970), validated with a permutation test with p-value 0.0071 <0.05), in the Lyon study.

Metabolite	Pathway
Isoleucine	BCAA
Valine	BCAA
Leucine	BCAA
α-keto-β-methylvalerate	BCAA

Table 4S. List of significantly changed baseline adjusted metabolites in males in the Lyon study and respective p-values calculated by t-test corrected for multiple comparison using Bonferroni-Dunn with α = 0.05 (n.s. = non-significant).

Metabolite	T0/T15 placebo	T0/T15 polyphenol	T0/T21 placebo	T0/T21 polyphenol	T0/T31 placebo	T0/T31 polyphenol
Glucose	n.s.	1.4×10^{-4}	n.s.	n.s.	n.s.	n.s.
Lactate	n.s.	7.3×10^{-3}	n.s.	1.6×10^{-2}	n.s.	n.s.
Citrate	2.5×10^{-2}	n.s.	n.s.	n.s.	n.s.	n.s.
Acetate	n.s.	n.s.	3.4×10^{-2}	n.s.	n.s.	n.s.
β-hydroxybutyrate	4.6×10^{-2}	9.6×10^{-3}	4.3×10^{-3}	8.5×10^{-8}	n.s.	n.s.
α-hydroxybutyrate	5.2×10^{-12}	1.6×10^{-7}	2.5×10^{-7}	3.3×10^{-3}	n.s.	n.s.
Alanine	n.s.	n.s.	n.s.	7.2×10^{-3}	n.s.	1.8×10^{-2}
Proline	n.s.	3.5×10^{-3}	n.s.	4.3×10^{-3}	n.s.	9.9×10^{-3}
Lysine	n.s.	9.7×10^{-3}	n.s.	n.s.	n.s.	n.s.
Valine	n.s.	n.s.	n.s.	n.s.	1.8×10^{-2}	n.s.
Leucine	2.1×10^{-2}	n.s.	n.s.	n.s.	2.6×10^{-3}	n.s.
Isoleucine	1.8×10^{-3}	n.s.	3.0×10^{-2}	n.s.	2.3×10^{-5}	n.s.
α-keto-isocaproate	4.2×10^{-2}	n.s.	n.s.	n.s.	n.s.	n.s.
α-ketobutyrate	2.8×10^{-2}	n.s.	1.6×10^{-2}	n.s.	n.s.	n.s.

Table 5S. Discriminant plasma metabolites after the exposure to 31 days of polyphenol and overfeeding (polyphenol, T31 versus T0) obtained by multi-level PLS-DA (model with a classification rate of 0.809 (95% CI of 0.659 and 0.914), validated with a permutation test with p-value 0.0203 <0.05) in the Lyon study.

Metabolite	Pathway
β-Hydroxybutyrate	Ketogenesis
Alanine	Glucose-alanine cycle

Table 6S. Lists of p-values of relative concentrations (not baseline adjusted), used in Fig 3B: placebo vs. Polyphenol for all the time points in the Lyon study; p-value >0.05 is considered as not significant (n.s.).

Metabolite	T0 placebo/ T0 polyphenol	T15 placebo/ T15 polyphenol	T21 placebo/ T21 polyphenol	T31 placebo/ T31 polyphenol
Valine	n.s.	n.s.	n.s.	1.5×10^{-2}
Leucine	n.s.	n.s.	n.s.	9.6×10^{-3}

Isoleucine	n.s.	n.s.	n.s.	3.6×10^{-3}
Glucose	n.s.	8.9×10^{-3}	3.4×10^{-2}	1.1×10^{-3}

Table 7S. List of significantly changed baseline adjusted metabolites, in females in the Lausanne study and respective p-values calculated by t-test corrected for multiple comparison using Bonferroni-Dunn with $\alpha = 0.05$ (n.s. = non-significant).

Metabolite	T0/T15 placebo	T0/T15 polyphenol	T0/T21 placebo	T0/T21 polyphenol	T0/T31 placebo	T0/T31 polyphenol
Lactate	n.s.	2.3×10^{-2}	n.s.	2.5×10^{-3}	n.s.	n.s.
Acetate	n.s.	n.s.	n.s.	1.7×10^{-2}	n.s.	n.s.
β-hydroxybutyrate	n.s.	4.8×10^{-2}	2.5×10^{-2}	4.6×10^{-3}	n.s.	n.s.
α-hydroxybutyrate	n.s.	3.5×10^{-9}	n.s.	2.3×10^{-6}	n.s.	n.s.
Proline	n.s.	7.1×10^{-3}	n.s.	4.0×10^{-5}	n.s.	n.s.

References

1. Wishart DS, Feunang YD, Marcu A, Guo AC, Liang K, Vázquez-Fresno R, Sajed T, Johnson D, Li C, Karu N, et al. HMDB 4.0: The human metabolome database for 2018. *Nucleic Acids Res* (2018) **46**:D608–D617. doi:10.1093/nar/gkx1089
2. Madrid-Gambin F, Oller-Moreno S, Fernandez L, Fernandez L, Bartova S, Giner MP, Joyce C, Ferraro F, Montoliu I, Moco S, et al. AlpsNMR: An R package for signal processing of fully untargeted NMR-based metabolomics. *Bioinformatics* (2020) **36**:2943–2945. doi:10.1093/bioinformatics/btaa022
3. Dieterle F, Ross A, Schlotterbeck G, Senn H. Probabilistic quotient normalization as robust method to account for dilution of complex biological mixtures. Application in1H NMR metabonomics. *Anal Chem* (2006) **78**:4281–4290. doi:10.1021/ac051632c
4. Filzmoser P, Liebmann B, Varmuza K. Repeated double cross validation. *J Chemom* (2009) **23**:160–171. doi:10.1002/cem.1225
5. Westerhuis JA, van Velzen EJJ, Hoefsloot HCJ, Smilde AK. Multivariate paired data analysis: Multilevel PLSDA versus OPLSDA. *Metabolomics* (2010) **6**:119–128. doi:10.1007/s11306-009-0185-z
6. Westerhuis JA, Brunius C, Rosén J, Shi L, Landberg R. Variable selection and validation in multivariate modelling. *Bioinformatics* (2018)1–9. doi:10.1093/bioinformatics/bty710
7. Segrestin B, Delage P, Nemeth A, Seyssel K, Disse E, Nazare J-A, Lambert-Porcheron S, Meiller L, Sauvinet V, Chanon S, et al. Polyphenol Supplementation Did Not Affect Insulin Sensitivity and Fat Deposition During One-Month Overfeeding in Randomized Placebo-Controlled Trials in Men and in Women. *Front Nutr* (2022) **9**:1–13. doi:10.3389/fnut.2022.854255
